# Gram Stains: A Resource for Retrospective Analysis of Bacterial Pathogens in Clinical Studies

**DOI:** 10.1371/journal.pone.0042898

**Published:** 2012-10-11

**Authors:** Usha Srinivasan, Sreelatha Ponnaluri, Lisa Villareal, Brenda Gillespie, Ai Wen, Arianna Miles, Brigette Bucholz, Carl F. Marrs, Ram K. Iyer, Dawn Misra, Betsy Foxman

**Affiliations:** 1 Department of Epidemiology, School of Public Health, University of Michigan, Ann Arbor, Michigan, United States of America; 2 Molecular Genetics Laboratory, Michigan Medical Genetics Laboratories, University of Michigan Medical Center, Ann Arbor, Michigan, United States of America; 3 Department of Biostatistics, School of Public Health, University of Michigan, Ann Arbor, Michigan, United States of America; 4 Department of Family Medicine, Wayne State University, Detroit, Michigan, United States of America; University of Iowa Carver College of Medicine, United States of America

## Abstract

We demonstrate the feasibility of using qPCR on DNA extracted from vaginal Gram stain slides to estimate the presence and relative abundance of specific bacterial pathogens. We first tested Gram stained slides spiked with a mix of 10^8^ cfu/ml of *Escherichia coli* and 10^5^ cfu/ml of *Lactobacillus acidophilus*. Primers were designed for amplification of total and species-specific bacterial DNA based on 16S ribosomal gene regions. Sample DNA was pre-amplified with nearly full length 16S rDNA ribosomal gene fragment, followed by quantitative PCR with genera and species-specific 16S rDNA primers. Pre-amplification PCR increased the bacterial amounts; relative proportions of *Escherichia coli* and *Lactobacillus* recovered from spiked slides remained unchanged. We applied this method to forty two archived Gram stained slides available from a clinical trial of cerclage in pregnant women at high risk of preterm birth. We found a high correlation between Nugent scores based on bacterial morphology of *Lactobacillus, Gardenerella and Mobiluncus* and amounts of quantitative PCR estimated genus specific DNA (*rrn* copies) from Gram stained slides. Testing of a convenience sample of eight paired vaginal swabs and Gram stains freshly collected from healthy women found similar qPCR generated estimates of *Lactobacillus* proportions from Gram stained slides and vaginal swabs. Archived Gram stained slides collected from large scale epidemiologic and clinical studies represent a valuable, untapped resource for research on the composition of bacterial communities that colonize human mucosal surfaces.

## Introduction

Technologies that identify bacteria based on DNA sequence rather than culture-based assays are dramatically changing our understanding of the bacteria colonizing human mucosal surfaces. Because numerous factors influence bacterial colonization and the variation in microbiota among individuals is large, to identify associations between commensal bacteria and human health and disease requires testing specimens from large numbers of individuals [1]–[3]. The greatest expense of such studies is obtaining the specimens. Therefore, if already collected material can be used, costs are greatly reduced.

The Gram stain is commonly used in epidemiologic and clinical studies; it involves smearing a sample onto a slide, staining the material using dyes that bind to bacterial cells, and visually inspecting under a microscope [4]. Many epidemiologic and clinical studies of pregnancy, sexually transmitted disease and lower respiratory infections include Gram stains in the study protocol [3]–[4]. Clinically, Gram stains are performed routinely to guide diagnosis and treatment [5]. When polymicrobial infections are suspected, as in bacterial vaginosis, Gram stains are scored based on bacterial morphologies without knowledge of the exact identities of the bacterial organisms present. For example, in the Nugent method of Gram stain classification, samples are analyzed for *Gardenerella*, *Mobiluncus* and *Lactobacillus* morphotypes and the resultant scores are used as a laboratory diagnostic for bacterial vaginosis [6]. Frequently, Gram stain slides are archived and re-scored. However, as we show here, Gram stains are a potential source of bacterial DNA; using quantitative PCR, Gram stains can be interrogated to identify the presence and amount of specific bacterial genera and species. We demonstrate the feasibility of extracting bacterial genomic DNA from archival vaginal Gram stain slides to get an estimate of presence and relative proportions of specific bacterial organisms using qPCR. We validated our methods by spiking slides with known quantities of *E.coli* and *Lactobacillus* and estimated recovery of bacterial organisms from stained and unstained slides using quantitative PCR. *Lactobacillus, Gardnerella* and *Mobiluncus* proportions were estimated from archival Gram stain slides using bacterial organism specific quantitative PCR and were found to be highly correlated to Nugent scores available from when the samples were initially collected. We also found similar proportions of *Lactobacillus* from paired vaginal swabs and Gram stains freshly collected from healthy women. Although qPCR validation is required before testing each organism of interest, , archived Gram stains can serve as a valuable resource for initial surveying of specific bacterial organisms and as preliminary resource to study bacterial community composition.

## Materials and Methods

### Sample testing

Our purpose was to determine if the DNA extracted from archived Gram stains would be useable for the identification of the presence and relative proportions of bacterial organisms of interest in clinical and epidemiologic studies. We extracted DNA from three different materials: 1) Glass slides spiked with known amounts of a mix of two bacterial organisms followed by comparison of recovery of DNA from Gram stained and unstained slides. 2) archived Gram stained slides with associated Nugent scores available from when samples were first collected for recovery of bacterial vaginosis associated bacteria; and 3) paired vaginal swab and Gram stained slides freshly collected from healthy women for the recovery of *Lactobacillus*.

We first tested our procedures on test slides spiked with known amounts of a mixture of *Lactobacillus* and *Escherichia coli*. Pure cultures of *E. coli* (strain MG1655) and *Lactobacillus acidophilus* (*L. acidophilus*, ATCC *832*) were grown overnight at 37°C on LB and MRS media, respectively. *E. coli* and *L. acidophilus* cell cultures were diluted to 10^8^ cfu/ml and 10^5^ cfu/ml respectively; 2 ml of each bacteria was mixed together and split into seven aliquots. From each aliquot 100 µl of the combined bacterial stock was added to paired microscopy glass slides. After heat fixing for 2 minutes, one slide from each pair was Gram stained using a commercially available kit (BD Diagnostics, MD) following manufacturer's instructions. The other slide from each pair underwent similar mock wash procedures as the Gram stained slides. After processing, the slides were allowed to air dry and stored at room temperature before DNA extraction and total bacterial DNA, *E.coli* and *Lactobacillus* were determined by qPCR.

To determine whether our protocol would work on archived slides that had been stored for 4 years we tested a subset of 42 archived vaginal slides collected from pregnant women with a range of Nugent scores, who participated in a randomized clinical trial on the use of cerclage to prevent preterm birth performed by a consortium of 15 US clinical centers between January 2003 and November 2007 [7]. The protocol and consent forms received local institutional review board approval at all centers. Gram stained slides, Nugent scores and pH of vaginal lavage were available for these women. After DNA extraction, amounts of total bacteria, *Lactobacillus, Gardenerella* and *Mobiluncus* were determined by qPCR. The analyses of already collected data were deemed exempt by the University of Michigan Institutional Review Board (UMIRB). The University of Michigan has provided a formal waiver for analysis of already collected data. All data are de-identified for anonymous analysis.

We also collected paired self collected vaginal swabs and vaginal smears on glass slides from eight healthy participants who participated in a pilot study for comparison of qPCR assays from fresh swab vs. Gram stained slides. Wayne State University IRB approval was obtained for this study. Written and signed informed consent was obtained from all participants. Slides were Gram stained by a clinical microbiologist. DNA extraction and qPCR analysis was performed on both Gram stained slide and vaginal swabs from each participant.

### DNA extraction

Total DNA from Gram stained and unstained vaginal slides were extracted using a commercially available kit (Puregene, Gentra, MN) with modifications. Briefly, slides were treated with xylene to remove immersion oil (used for Gram staining) followed by treatment with 1∶1 xylene∶ethanol to prevent re-precipitation of crystal violet/safranin. After a final rinse in 100% ethanol, slides were air dried and the vaginal smear was treated with 75 µl cell lysis buffer (Qiagen DNeasy kit) and the vaginal sample was lifted off the slide and transferred to a eppendorf tube containing 16 µl of 25 mg/ml lysozyme and 4 µl of 20 ug/ml lysostaphin. After incubation at 37°C for 30 minutes, the lysate was treated with 4 µl of 4 mg/ml RNase A for 10 minutes at 37°C and total DNA extracted in a Qiagen M48 automated system using manufacturer's recommendations. For the vaginal swab, a similar method was followed without treatment with xylene and ethanol rinse. Purified DNA samples were stored at −20°C.

### Quantitative PCR using universal bacterial primers

Quantitative PCR was used to quantitate total genomic DNA extracted from Gram stained slides using the SYBR green fluorescent detection system. Total bacterial load was determined with a broad range universal primer set based on the 16s rDNA region from the domain Bacteria. A number of universal primer sets for bacterial genomic DNA detection have been reported in literature; however none of the universal bacterial primer sets available afford complete coverage of all bacterial taxa [8]. We used the universal primer set published by Nadkarni et. al. [9] because taxon coverage from this primer set is as good or better than 8 other published universal primer sets [10]. Performance, optimal annealing temperatures and expected product sizes for qPCR assays for *E. coli, Lactobacillus, Gardnerella* and *Mobiluncus* were initially confirmed by traditional PCR (PE thermal cycler 9700) followed by assay optimization in qPCR (Biorad CFX96). Quantitative PCR reactions were carried out in a total volume of 20 µl, using a commercially available SYBR green PCR master mix (Ssofast EvaGreen Supermix, Bio-Rad, CA) and 200 nM universal primers. SYBR green real time PCR amplification conditions included an initial denaturation step at 98°C for 2 minutes to release the Sso7d-fusion antibody originally bound to the polymerase. This was followed by 40 cycles of 98°C for 4 seconds and 60°C for 4 seconds. Standard curves for determining total bacteria from vaginal slides were generated from qPCR reactions using a 10-fold dilution series of the cloned 16S rDNA insert from the different bacteria (*Lactobacillus, Gardnerella, Mobiluncus, E. coli*) and determined to be linear between 10^2^ *rrn* copies and 10^8^ *rrn* copies. Melt curve analyses were carried out in conjunction with each real time PCR assay (melt temperature for bacterial qPCR assays: *Mobiluncus*: 81°C, *Lactobacillus*: 84C, universal bacterial primer: 85°C, *Gardnerella*: 87°C and *E. coli*: 83°C;) to distinguish fluorescent signal from primer specific amplification product from non-specific primer dimer formations. Assay conditions were optimized to reduce the amount of primer dimer in order to maximize the bacteria specific signal in the qPCR assay.

### Pre-amplification using 16 S rDNA gene

The total amounts of bacterial DNA isolated from archived slides were in the range 10^3^–10^4^ *rrn* copies/µl. A bacterial taxa present at 10% abundance would only yield 10^1^–10^2^ *rrn* copies/µl template after a 10 fold dilution in the qPCR assay which is close to the limit of detection in the qPCR assay. Therefore, pre-amplification was performed to increase the amount of DNA template available for bacterial taxa identification and quantitation. Pre amplification for a nearly full length 16S rDNA gene fragment (1484 bp) was optimized using bacterial genomic DNA from *E.coli* and *Lactobacillus*. Briefly, PCR was carried out with an initial denaturation step at 98°C for 2 minutes, then followed by 8, 10, or 12 cycles of 98°C for 30 seconds and 60°C for 1 minute 30 seconds using the 8F forward (5′-AGAGTTTGATCCTTGGCTCAG-3′) and 1492R reverse primers (5′-GCYTACCTTGTTACGACTT-3′) (5). Pre-amplification reactions using different cycles were performed on the CFX-96 thermal cycler (Bio-Rad, CA) to determine the optimum number of cycles that would yield the highest signal in qPCR assay without introducing significant PCR bias.

### Quantitative PCR assay for *Lactobacillus*, *Escherichia coli*, *Gardnerella and Mobiluncus*

Primers specific to the 16S ribosomal genes (*rrn*) in *Lactobacilli*, *Escherichia coli. Gardenerella and Mobiluncus* were designed using primer design software (AlleleID, v.7, Premier Biosoft International) using 16S rDNA sequences available in NCBI and RDP (http://www.ncbi.nlm.nih.gov) (Table 1). The *Lactobacillus* primers were determined to be specific for *L. acidophilus, L. delbrueckii, L. gasseri, L. crispatus, L. helveticus, L. jensenii, L. johnsonii, L. bulgaricus*, and *L. murinus* by *in silico* analysis with available sequences in NCBI. Quantitative PCR was performed in a CFX96 Thermocycler (Bio-Rad, CA) and annealing conditions were optimized (98°C for 2 min. followed by 40 cycles of 98°C for 4 sec. and 63°C for 4 sec.) using purified genomic DNA from *L. acidophilus* as positive control and a mix of bacterial genera as negative control. *E. coli* conditions were optimized using strain MG1655 as a positive control and the annealing conditions 98°C for 2 min. followed by 40 cycles of 98°C for 1 sec. and 58°C for 1 sec. *Gardenerella* primers were specific for *G. vaginalis* and *Mobiluncus* primers were specific to *M.curtsii* and *M. mulieris*. Genomic DNA purified from twelve bacterial organisms from nine genera that are reported to reside in the vaginal sites were purchased from ATCC (*Lactobacillus, Fusobacterium, Veillonella, Porphyromonas, Prevotella, Campylobacter, Mobiluncus, Tannerella, Atopobium*, *Gardnerella*) as well as *Escherichia coli* (strain MG1655) were included in the appropriate controls for PCR to ensure that primers and PCR conditions were optimized for *Lactobacillus* genera and *E. coli*. The 16S rDNA gene fragment obtained from PCR was cloned into a commercial vector (TOPO TA cloning kit, Invitrogen, CA). The limit of detection was 10^2^ *rrn* copies for total bacteria, *E. coli*, *Lactobacillus, Gardnerella* and *Mobiluncus*. All assays were conducted in triplicate and the mean values used for analysis; r^2^ values ranged from 0.98 to 0.99.

**Table 1:** Primers used for quantitation of total bacteiral DNA, *Lactobacillus* and *E. coli*.

Primer	Sequence	Primer concentration	Fragment Size	Reference
Preamp primers(8F/1492R)	F: 5′AGAGTTTGATCCTTGGCTCAG-3′R: 5′-GCYTACCTTGTTACGACTT-3′	500 nM	1484 bp	(5, 13)
Universal bacterial primers	F: 5′-TCCTACGGGAGGCAGCAGT-3′R: 5′-CGACTACCAGGGTATCTAATCCTGTT-3′	200 nM	445–466 bp	9
*Lactobacilli*(LacF/LacR)	F: 5′-AGGAGAGTGGAACTCCATGTG-3′R: 5′-ACRGCTTTAAGAGATYYGCTWR-3′	750 nM	648 bp	This study
*E. coli*(ecoli_16S/464f/ecoli_16S_567r)	F: 5′-TAATACCTTTGCTCATTG-3′R: 5′-CCAGTAATTCCGATTAAC-3′	500 nM	103 bp	This study
*Gardenerella*	F: 5′-GCCTGACGACTGCAGAGATGT-3′R: 5′-ATTAGCACCATGTCACCATGAAG-3′	500 nM	282 bp	This study
*Mobiluncus*	F: 5′-CGGATTTATTGGGCGTAA-3′R: 5′-GCAGACCAACAGTTAAGC-3′	500 nM	79 bp	This study

In a separate experiment, we compared the *Lactobacillus* proportions obtained using the 628 bp *Lactobacilli* 16S rDNA fragment used in this study to that obtained from qPCR assay using a smaller sized 16S rDNA fragment (325 bp). *Lactobacillus* proportions identified using the two assays were similar (data not shown); suggesting that genomic DNA fragmentation in archived samples does not significantly affect the *Lactobacillus* results from qPCR. We also tested serial dilutions of the DNA extracted from Gram stained material (both freshly spiked as well as archived slides); serial dilutions gave the expected Ct values in qPCR (data not shown) and we ruled out inhibition of qPCR as a potential source of error for DNA extracted from Gram stained slides.

### Analysis

Log_10_ transformed total bacterial DNA 16s *rrn* copies extracted from spiked unstained and Gram stained slides were analyzed. We used the Student t-test to test for differences in the mean log _10_ total bacterial 16S rrn copies and relative log *Lactobacilli rrn* copies (normalized to total bacterial DNA) between paired stained and unstained slides after spiking. The effects of different pre-amplification cycle numbers on 16S *rrn* recovered from stained and unstained slides were also determined. For archived slides, we used Kruskal Wallis tests to determine significant difference in the quantitative proportions of bacterial vaginosis bacteria and Nugent scores. For the comparison of *Lactobacillus* proportions from vaginal swab vs Gram stained slides data were fitted to linear mixed model followed by post hoc analysis using Tukey. All analysis was performed using SAS 9.0 (SAS Institute, NC) or R software.

## Results

### Spiked slides

**Figure 1:**
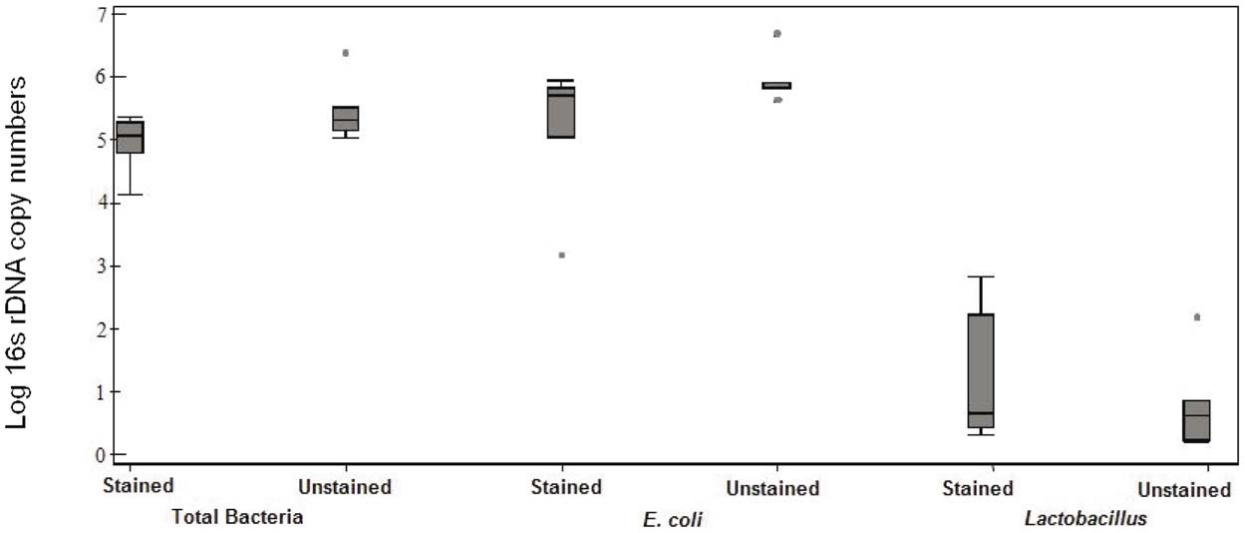
Total bacterial DNA, total *Escherichia coli* and total *Lactobacillus* 16S rDNA copy numbers from Gram stained and unstained slides spiked with *Escherichia coli* and *Lactobacillus*. Box plot represent the observed values for seven slides for stained and unstained slides, individual spots represent outliers. Slides were spiked with a 1∶1 mix of 10?8 and 10?5 cfu/ml of *E.coli* and *Lactobacillus* respectively. For each pair one replicate was Gram stained while the other was left unstained. After DNA extraction from stained and unstained slides, samples were analyzed for 16S rDNA copies using species and genera specific quantitative PCR.

**Figure 2:**
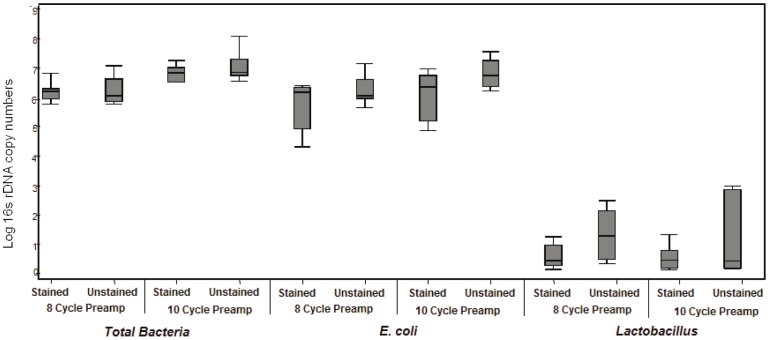
Total bacterial DNA, total *Escherichia coli* and total *Lactobacillus* 16S rDNA copy numbers from DNA extracted from seven Gram stained and unstained slides spiked with *Escherichia coli* and *Lactobacillus*. Slides were spiked with mixture of mixture of 10?8 and 10?5 cfu/ml of *E.coli* and *Lactobacillus*. For each pair one replicate was Gram stained while the other was left unstained. After DNA extraction from stained and unstained slides, samples were preamplified using 8F-1492R degenerate bacterial primers for different cycles and analyzed for 16S rDNA copies using species and genera specific quantitative PCR.

As proof of principle, we determined if the process of Gram staining affects the recovery of total bacterial DNA from slides spiked with known amounts of *E. coli* and *Lactobacillus*; we did not find significant differences in the relative proportions of *E. coli* and *Lactobacillus* from paired stained and unstained slides (Figure 1). We used a nested qPCR approach with pre-amplification using 8F-1492R universal bacterial primers to amplify a 1484 bp fragment of the bacterial 16S ribosomal gene. The pre-amplification was followed by qPCR with 16S rDNA primers specific to *E. coli* and *Lactobacilli* or total bacteria (Table 1). Cycle numbers for the nested pre-amplification steps were set lower than the threshold for detecting signal in the qPCR assay to reduce PCR bias. Three different pre-amplification conditions were tested; 8 cycles, 10 cycles and 12 cycles followed by qPCR with universal bacterial primers, *E. coli* and *Lactobacilli* specific primers. Pre-amplification at 8 and 10 cycles did not introduce a detectable bias in the levels of *E. coli* or of *Lactobacilli* detected using qPCR, since a) the total bacteria, total *Lactobacilli* and total *E. coli* increased proportionally with increasing number of pre-amplification cycles (data not shown) and b) the relative amounts of *Lactobacilli* and *E. coli* recovered under different pre-amplification conditions remained unchanged (Figure 2) and were similar to *Lactobacilli* and *E. coli* recovered from the original sample (no pre-amplification) (Figure 2 vs. Figure 1). Pre-amplification using 12 cycles was not pursued as the relative proportions of *E. coli* showed a tendency to increase compared to relative proportions of *E. coli* from samples without pre-amplification (data not shown). Pre-amplification is valuable for testing multiple bacterial taxa when starting template is limited, however different pre-amplification conditions will need to be tested to minimize amplification bias.

### Archival slides

**Figure 3:**
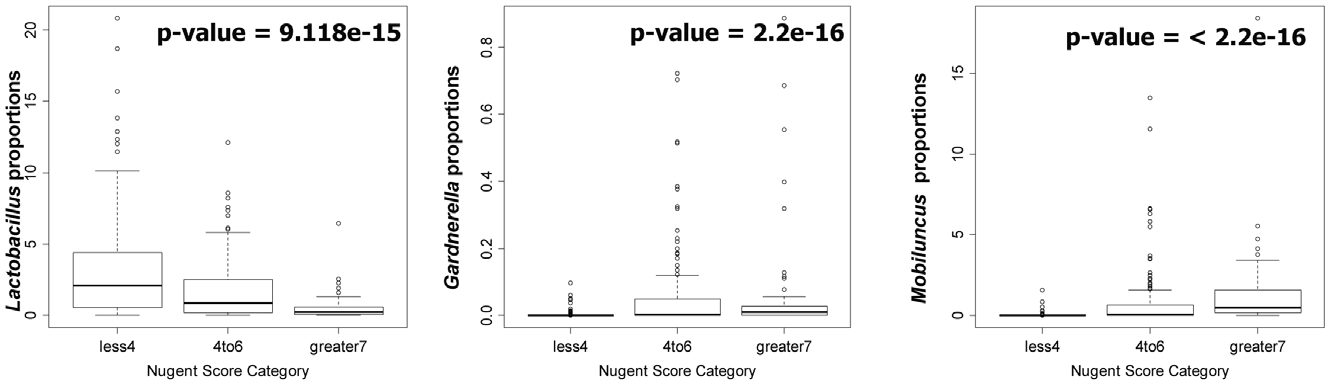
Correlation of qPCR assay for Lactobacillus, Gardnerella and Mobiluncus detected in DNA extracted from archived Gram stain slides and Bacterial vaginosis determined using Nugent Score. Gram stained slides were selected for 42 pregnant women enrolled in the Cerclage study for PTB who had a range of Nugent score from 0 to 10. Nugent score of 0–3 is classified as no BV, 4–6 is intermediate BV and >7 as BV. Nugent scores were determined by clinical technologists at the time of sample collection. qPCR assays were performed in triplicate for 16S rDNA copies of *Lactobacillus*, *Gardnerella* and *Mobiluncus* and normalized with total bacterial 16S rDNA determined using universal bacterial primers. Kruskal Wallis p-values showed significant association of *Lactobacillus, Gardnerella* and *Mobiluncus* with bacterial vaginosis (p<0.01).

We applied the nested qPCR approach to Gram stained slides from forty-two women, collected in 2003–2007 from pregnant women enrolled in the Vaginal Ultrasound Trial Consortium [7]. *Lactobacilli* are normal inhabitants of the healthy vaginal mucosa; *Gardenerella* and *Mobiluncus* proportions are often found raised in bacterial vaginosis although they can be detected in lower levels in healthy vagina as well [6]. Nugent scores for *Lactobacillus, Gardnerella* and *Mobiuncus* numbers (morphology based) were available from when the samples were collected. We therefore targeted *Lactobacillus, Gardnerella* and *Mobiluncus* in these archived slides for qPCR following pre-amplification. Genomic DNA was extracted from slides and the 16S *rrn* copies for *Lactobacilli*, *Gardnerella* and *Mobiluncus* were determined using bacteria specific qPCR assays; total bacteria was estimated using the universal bacteria qPCR assay. Since the vaginal smears were self collected and no effort was made to standardize the amount of material deposited on the slides the total bacteria on replicate slides across individuals are different. Therefore we compared the recovery of relative proportions *Lactobacilli* from each individual slide (normalized to total bacterial DNA) rather than absolute amounts. Nugent scores were available for the slides from time of collection and ranged from 0 to 10. Nugent scores in the range 0–3 are considered to be BV negative and found in *Lactobacillus* dominated bacterial communities, Nugent 4–6 is intermediate BV and Nugent scores 7–10 are considered to be BV positive and associated with high levels of *Gardnerella* and *Mobiluncus* and low or absent *Lactobacillus*. Kruskal Wallis non parametric analysis was performed to determine the association of quantitative PCR estimated relative proportions of bacterial vaginosis bacteria (*Lactobacillus, Gardnerella* and *Mobiluncus*) and Nugent score. The proportions of BV bacteria determined using qPCR was significantly correlated with Nugent scores for BV (Figure 3).

### Comparison of vaginal swab with stained slides

Vaginal swabs are the standard method for sample collection from vaginal sites for bacterial community analysis. We compared qPCR generated results for proportions of specific bacteria from Gram stained slides to those obtained from vaginal swabs. Since we did not have archived vaginal swabs at our disposal we tested DNA extracted from paired vaginal swabs and Gram stained slides freshly obtained from 8 otherwise healthy women without BV. Total genomic DNA was extracted from swab and stained slide using the similar DNA extraction protocol; *Lactobacillus* proportions were determined using bacteria specific qPCR and total bacterial DNA using universal bacterial primers. The *Lactobacillus* proportions obtained from stained slides were not significantly different from those obtained from the swabs; pre-amplification of DNA extracted from stained slides did not significantly change the proportion of *Lactobacillus* detected (Table 2). We observed that pre-amplification of vaginal swab DNA resulted in increased estimates of *Lactobacillus* proportions compared to Gram stained slides (data not shown). Typically, swabs hold more material than slides; bacterial DNA extracted from swab is ∼100 fold higher than that obtained from slides (data not shown) and this could potentially introduce amplification bias during pre-amplification of swab DNA. Our results suggest that pre-amplification methods designed in this study are not indicated for vaginal swabs.

**Table 2:** Proportions of *Lactobacillus* recovered from genomic DNA extracted from paired Gram stained slide and vaginal swabs collected from eight healthy women.

Sample	Preamplification*	Average *Lactobacillus* proportion	*p* value**
Vaginal Swab (n = 8)	No	2.7±2.5	-
Gram stained slide (n = 8)	Yes	3.5±2.8	0.962
	No	3.2±1.5	0.999

* Preamplification was performed at 98°C for 2 minutes, then followed by 10 cycles at 98°C for 30 seconds and 60°C for 1 minute 30 seconds for 10 cycles using degenerate primers 8F-1492R targetting the bacterial 16S ribosomal gene.

** In comparison to average *Lactobacillus* proportions obtained from vaginal swab (n = 8) without pre amplification.

All assays were run in triplicate.

## Discussion

We demonstrate that DNA extracted from Gram stained slides is potentially amenable to molecular analysis for detection of bacterial pathogens using qPCR. Firstly, when we used a mix of *E.coli* and *Lactobacillus* for Gram staining, we were able to obtain the expected qPCR signal for *E.coli* and *Lactobacillus* in proportion to the relative amounts of initial amounts used for spiking. Note that 1000 fold higher colony forming units of *E.col*i was used compared to *Lactobacillus* for spiking with a mix of *E.coli* and *Lactobacillus*; as expected higher *E. coli* 16S *rrn* copies were recovered in comparison to *Lactobacillus* after extraction of DNA, pre-amplification and qPCR. However, due to differences in the *rrn* copy numbers of *E. coli* and *Lactobacillus*, the ratio of qPCR generated *Lactobacillus* and *E. coli* concentrations is not equal to the 1000 fold difference in their initial spike concentrations. Secondly, for archival slides, qPCR estimated relative proportions of *Lactobacillus*, *Mobiluncus* and *Gardnerella* after pre-amplification of total bacterial DNA were significantly correlated with the previously determined Nugent score for BV diagnosis. qPCR generated proportions of *Lactobacilli*, *Gardenerella* and *Mobiluncus* from genomic DNA extracted from Gram stained slides appear to be robust to variations in total bacterial DNA present on the slides, indicating that DNA extracted from archived Gram stain slides is suitable for estimating both frequency and proportions of specific bacterial organisms. There has been one previous case report which used PCR to detect specific bacteria from a fresh Gram stain when culture results from the clinical specimen was negative [11]. To our knowledge, our work is the first study to successfully use archived Gram stained slides to determine relative proportions of specific bacteria although similar methods have been used to quantify *Cryptosporidium* in oocytes from fecal slides [12] and for recovery of human DNA in forensic samples [13]. Our results indicate that the effect of Gram staining on the proportions of *Lactobacillus*, *Gardnerella* and *Mobiluncus* obtained by qPCR from Gram stained slides in comparison to the swab is not statistically significant. Vaginal swabs remain the standard method of sample collection for quantitation of specific bacteria in human samples, our results show that DNA from Gram stained slide can nevertheless be used as a first step for semi-quantitative estimate of relative proportions of specific bacteria present.

While we were able to demonstrate the usefulness of this method for a select number of bacterial species, we were limited by our use of 16S ribosomal gene based phylogenies for bacterial identification. Total bacterial DNA was estimated using a “universal” primer, which varies in completeness of coverage of different genera present in the vaginal samples. We chose primers identified in the literature as most appropriate for vaginal samples, but as new bacterial species are discovered the universality of these primers needs to be re-validated [8]. For the primers specific to *Lactobacillus* we tested a set of *Lactobacillus* species for which strains were available; however there is no one universal *Lactobacillus* genera primer and we cannot completely rule out non-specific amplification in the qPCR assay. Although our results compared well with the Nugent score, the Nugent score has its own limitations. *Lactobacilli* morphology can vary between the classic rods and cocci shapes [14], [15] and Gram stain identification for *Lactobacilli* may miss some of the *Lactobacilli* types present in the specimen. Extending the qPCR analysis of DNA Grams stained material to other bacteria found in the vaginal milieu will be an important next step to establish the taxa specific differences in stability over time for other commonly found taxa in human vaginal milieu.

In the qPCR assays on archival slides, *Lactobacillus* proportions greater than the theoretical maximum of 1 were found for many of the sample DNA extracted from both vaginal swab material as well as stained slides. Pre amplification does not appear to contribute significantly, as >1 Lactobacillus proportions were calculated both for samples that pre-amplified and those that were not. The high proportions obtained may be due to an underestimate of *Lactobacillus* genera by the universal bacterial primer or an overestimate of *Lactobacillus* 16S copies by the *Lactobacillus* assays, or both. The differences in 16S ribosomal copy numbers for *Lactobacillus* versus other bacteria present in the vaginal sample may also contribute; *Lactobacilli* typically have between 1–9 ribosomal operons/genome (average = 5.3); other bacterial genomes vary between 1 to 15 copies (http://rrndb.cme.msu.edu). However, we are unable to correct for differences in copy numbers in the calculation of the *Lactobacilli* proportion since we do not know the identity of all bacterial organisms that contribute to the qPCR signal in the universal bacterial assay. We observed a range of *Lactobacillus* proportions; ranging from 3.3 to 22 in genomic DNA extracted from pure cultures of different *Lactobacillus* species, when tested individually in the *Lactobacillus* qPCR assay (Table S1). Since the *Lactobacillus* qPCR assay is optimized for the Lactobacillus species and not in the universal bacteria assay, higher *Lactobacillus* proportions are likely due to the lower efficiency with which universal bacterial primers amplify *Lactobacillus* species or a subset of *Lactobacillus* species. Based on these findings, we suggest that the proportions of bacterial organisms obtained using qPCR of DNA from Gram stained material is at best a semi quantitative estimate of the true *Lactobacillus* proportion present in the sample. We were unable to quantitatively determine whether bacterial taxa in archived samples differ substantially in their temporal stability since qPCR was not performed on samples immediately after collection. However qPCR results suggest this is likely since Nugent scores available from when the study was conducted are found to be highly correlated to the recently performed qPCR assay for vaginal bacteria from archived slides.

The use of archived slides for hypothesis testing is a feasible alternative when vaginal swab material is otherwise unavailable or for generating data from previously conducted studies in order to generate new hypothesis that can then be tested using vaginal swab material. Ongoing studies in our laboratory are focused on developing and testing vaginal Gram stains for multiple bacterial taxa that are linked to human health and disease [16]. Many archival slides have relevant host-associated information that can be incorporated to further analyze host- bacterial dynamics in disease. DNA extracted from archival slides is not restricted to bacteria identification; fungi and viral organisms can also be studied. Preventing the introduction of additional amplification bias during pre-amplification allows the use of archival Gram stains in research and offers a promising resource to expand our understanding of the role of bacteria in human health and disease.

## Supporting Information

Table S1 *Lactobacillus* proportions from genomic DNA extracted from pure cultures of different *Lactobacillus* species, and tested individually in the *Lactobacillus* qPCR. *Lactobacillus* strains were cultured overnight in MRS broth and genomic DNA was extracted. All sample DNA were tested in *Lactobacillus* qPCR assay and universal bacterial qPCR assay. Samples were run in triplicate and mean values presented.
(XLSX)
